# Incarcerated Umbilical Hernia Treated With Adhesive Strapping in a Three-Month-Old Infant

**DOI:** 10.7759/cureus.40590

**Published:** 2023-06-18

**Authors:** Masazumi Miyahara, Kyoko Osaki

**Affiliations:** 1 Department of Pediatrics, Okanami General Hospital, Iga, JPN

**Keywords:** umbilical hernia, incarceration, ileus, children, adhesive strapping

## Abstract

An umbilical hernia (UH) is a common condition in early childhood; it is defined as the protrusion of abdominal viscera through a defect in the umbilical ring. Since most UHs close spontaneously, almost no active treatment has been performed. Adhesive strapping (AS) for early UH closure is an easy-to-perform and relatively safe treatment. However, it can present rare but serious complications. Herein, we report a case of a three-month-old infant with incarcerated UH during AS therapy. AS treatment for UH, which has been reported mainly in Japan, has shown excellent results in observational studies and could be widely applied globally. However, our case demonstrated the presence of a noteworthy complication; incarcerated UH, in addition to skin complications, such as cellulitis and skin ulcer-related perforation, was associated with AS. To minimize the occurrence of these complications associated with AS treatment for UH, it is crucial to adequately explain and guide the family members regarding the proper management of AS and seeking medical care promptly when abnormalities occur during AS.

## Introduction

Pediatric umbilical hernia (UH) is common, and the majority close spontaneously. No formal practice guidelines exist regarding the optimal timing and indications for repair [1]. In 1950, adhesive strapping (AS) was widely used as a treatment for UH [2]. However, after it was reported that a high proportion of UH close spontaneously, a wait-and-see policy was gradually adopted, and AS was abandoned. Almost 60 years later, AS for UH was reassessed in Japan. Since the early 2000s, AS for UH has been positively recommended for earlier UH closure and a reduction in redundant skin of UH [3]. Additionally, a few observational studies have shown its excellent efficacy [4,5]. Moreover, AS for UH has been approved by the Japanese health insurance system since 2014; therefore, the number of facilities performing AS for UH has been increasing in Japan [3]. However, AS has not been widely used for the treatment of UH. AS is considered to have no beneficial effect in Europe and America, and observation without any treatment is generally recommended for the management of UHs in infants [5]. Furthermore, there have been recent reports of severe complications associated with AS, such as cellulitis [6], skin ulcer-related perforation, and incarceration of UH in Japan. Among these, incarceration requires urgent diagnosis and treatment. Herein, we report a case of an infant with incarcerated UH during AS.

## Case presentation

A full-term three-month-old male infant was admitted to our hospital with recurrent vomiting. He had been treated with AS for UH when he was two months old. AS was performed as follows. Following the ease of the UH, its contents were repositioned into the abdominal cavity, with a corresponding volume of cotton wool introduced to maintain the hernia sac inverted within the abdomen. Post placement of the cotton wool, adjacent skin folds were approximated (aligning two rectus muscles in the center), thereby creating a single crease to conceal the sponge, all while applying an elastic adhesive strip to inhibit hernia protrusion. Ultimately, a protective dressing tape was layered over the adhesive strip to confer water resistance. The film dressing and adhesive strip were replaced weekly by the parents. No abnormalities in the site of AS were observed before the onset of symptoms. Two hours before the onset of vomiting, AS was temporarily released for bathing. After bathing, a protruding UH was observed, and the parents could not return it. The patient subsequently developed vomiting. On admission, his vital signs were as follows: temperature, 36.5 °C; pulse, 170 beats per minute; and blood pressure, 92/48 mmHg. The patient had a slightly pallid complexion and was lethargic. The abdomen was noticeably distended and stiff. The bowel sounds were diminished. UH prolapse was observed. The hernial orifice was 18 × 16 mm in diameter.

Laboratory analysis revealed the following: white blood cell count, 14,400 cells/µL (48% neutrophils); C-reactive protein level, 0.02 mg/dL; aspartate aminotransferase level, 31 IU/L; alanine aminotransferase level, 34 IU/L; blood urea nitrogen level, 11.1 mg/dL; creatinine level, 0.21 mg/dL; sodium level, 138 mEq/L; potassium level, 5.1 mEq/L; and glucose level, 109 mg/dL. Additionally, venous blood gas analysis showed a pH of 7.407, pCO2 of 37.3 mmHg, HCO3 of 23.1 mmHg, and base excess of -1.4 mmol/L (Table 1).

**Table 1:** Laboratory tests data on admission WBC: white blood cell count; RBC: red blood cell count; ALP: alkaline phosphatase; AST: aspartate aminotransferase; ALT: alanine aminotransferase; LDH: lactate dehydrogenase; CPK: creatine phosphokinase; T-Cho: total cholesterol; BUN: blood urea nitrogen; Crea: creatinine; CRP: C-reactive protein; pH: potential hydrogen; pCO2: carbon dioxide partial pressure; HCO3: bicarbonate; BE: base excess

Laboratory parameters	Patient value	Reference range
Peripheral blood test	On admission	
WBC	14400/μL	4000-9000
neutrophil	48%	39-81
lymphocyte	46%	16-50
monocyte	6%	2-10
RBC	426 × 10^4 ^/μL	400-520 × 10^4^
Hemoglobin	11.6 g/dL	13.0-17.0
Hematocrit	34.8%	38.0-49.0
Platelet	67.3 × 10^4 ^/μL	12.0-44.0 × 10^4^
Serum biochemical test		
Total protein	6.7 g/dL	6.5-8.5
Albumin	4.8 g/dL	4.1-5.3
Total bilirubin	0.18 mg/dL	0.20-1.20
glucose	109 mg/dL	70-109
ALP	255 U/L	38-113
AST	31 IU/L	10-35
ALT	34 IU/L	10-35
LDH	283 U/L	110-225
CPK	135 IU/L	50-200
T-Cho	175 mg/dl	150-219
BUN	11.1 mg/dL	9.0-22.0
Crea	0.21 mg/dL	0.50-1.10
Sodium	138 mEq/L	138-145
Potassium	5.1 mEq/L	3.4-4.7
Chloride	103 mEq/L	99-108
CRP	0.02 mg/dL	0.00-0.30
venous blood gas test		
pH	7.407 mmHg	7.320-7.410
pCO2	37.3 mmHg	41.0-51.0
HCO3	23.1 mmHg	24.0-28.0
BE	-1.4 mmol/L	±3

**Figure 1:**
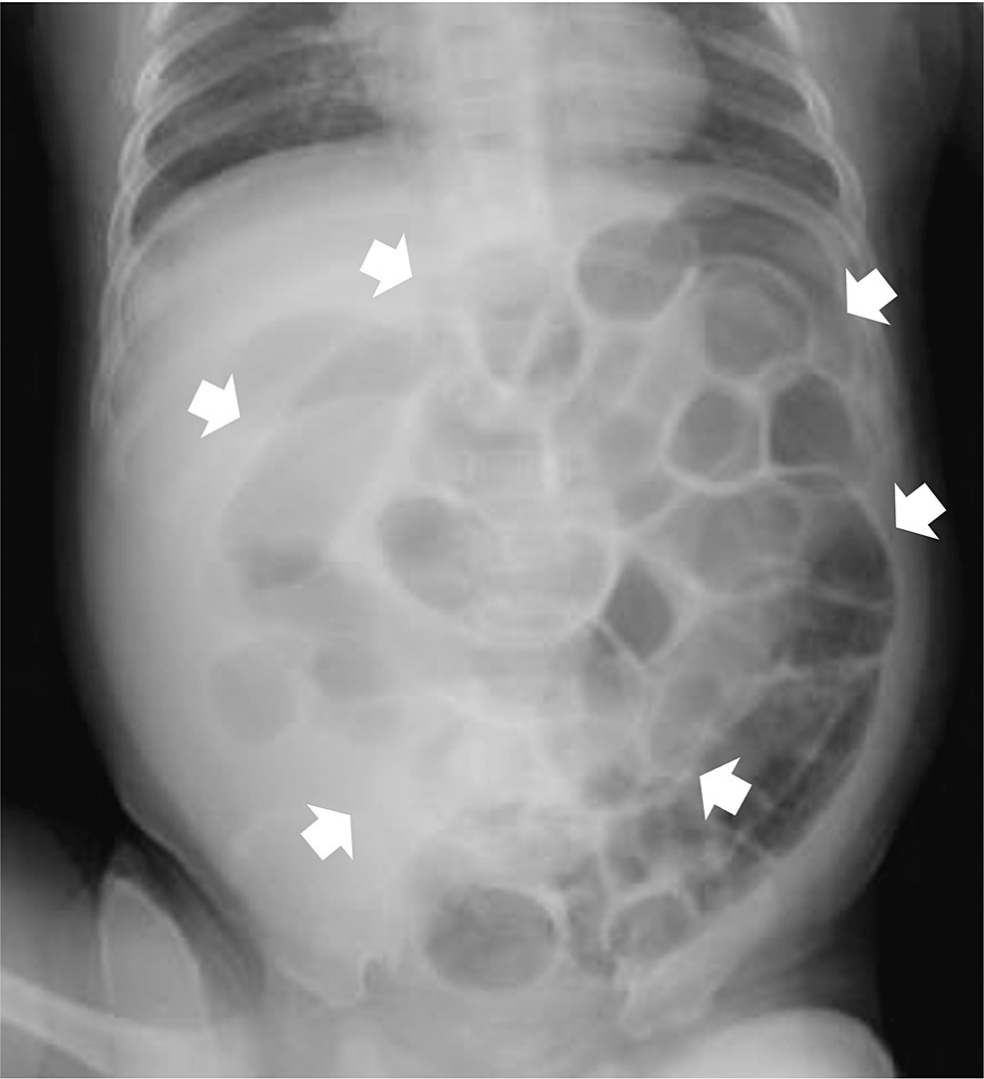
Abdominal radiograph at the spine position showing an extensive dilated small intestine

**Figure 2:**
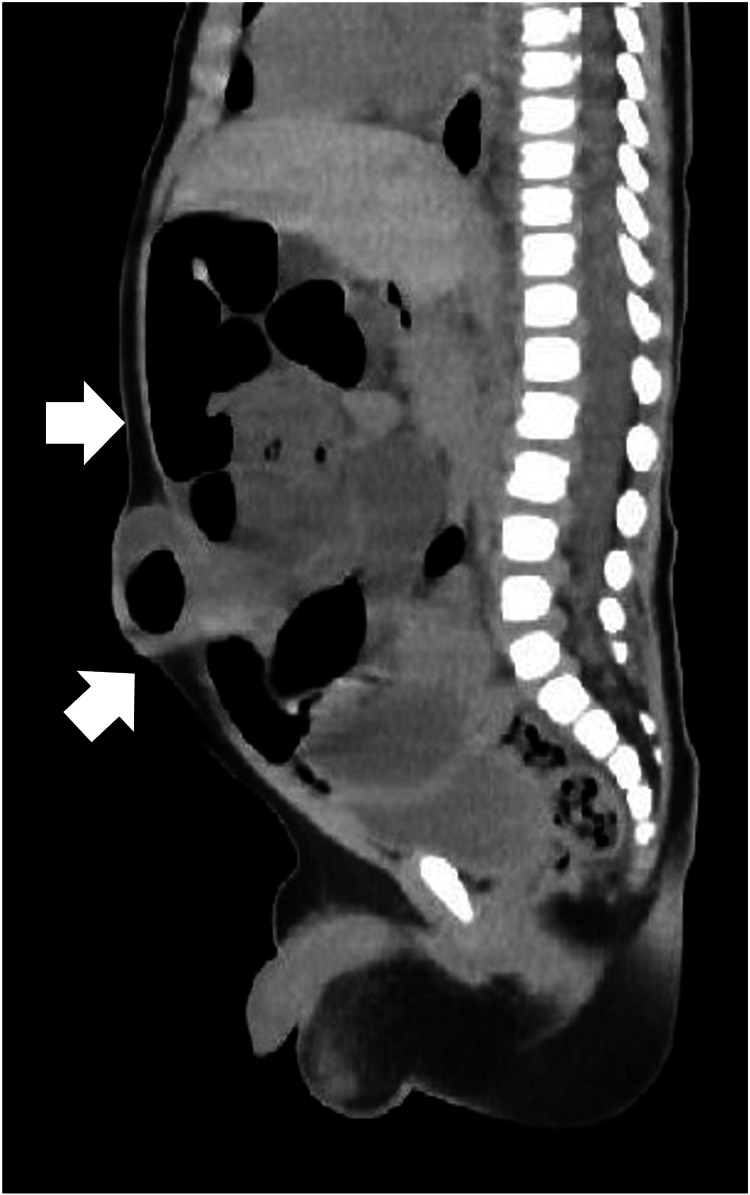
Abdominal computed tomography showing the prolapse of the UH with dilated intestines

Abdominal radiography revealed extensive dilation of the small intestine, indicating obstructive ileus (Figure 1). Abdominal computed tomography further showed bowel obstruction with prolapse of the UH (Figure 2). We successfully attempted manual reduction of the UH without using any sedation. Specifically, using digital manipulation, the protruded intestine was manually reduced into the hernia orifice. Despite encountering slight resistance, a successful reduction was accomplished. Shortly thereafter, the abdominal fullness disappeared, and the patient became comfortable. Six hours after manual reduction, milk feeding was resumed with no complications, and the patient was discharged the next day. The patient continued to receive AS for UH after discharge, and at four months of age, the UH resolved and AS was discontinued simultaneously.

## Discussion

In our case, intestinal obstructive ileus due to prolapse of UH treated during AS occurred, which worsened the patient’s general condition. Therefore, it can be judged as an incarcerated UH. Since the first report of incarcerated hernia in a Japanese infant in 2015 treated with AS, five cases, including ours, have been reported. Except for our case, all other cases have been reported in the Japanese literature. Therefore, regrettably, those reports are not listed in PubMed. According to these reports, in two cases of incarceration, manual reduction was impossible, and emergency surgery was performed. Moreover, in three cases, the material used in AS was pushed out from the site where it was placed during AS owing to abdominal pressure. Therefore, the intestinal tract prolapsed beyond the narrowed site and incarcerated. In all cases, except our case, the time from the onset of abdominal symptoms to consultation exceeded 12 hours. In contrast, in our case, the time from the onset to the consultation was approximately two hours; hence, the manual reduction was successful, and the subsequent course was favorable.

The incarceration occurred within almost 20 minutes after the complete release of the AS. The event had not occurred during AS, no dermatitis or ulceration was observed on the skin at the site of AS, and the AS-related procedures performed were completely in accordance with those commonly practiced in Japan. Therefore, AS for UH was considered to have been performed appropriately. Although the initial size of the hernia orifice was unknown, the main cause of hernia incarceration in our case could have been the rapid narrowing of the hernia orifice due to the effect of the AS, which can be an iatrogenic complication. Our patient was able to complete the AS therapy only for approximately two months because of achieving no UH prolapse. Additionally, the early and complete healing of his UH can support the possibility of the rapid narrowing of the patient’s hernia orifice during AS treatment. However, incarceration can further occur in UHs without AS. The overall risk for incarceration of a pediatric UH without AS is estimated to be 0.07-0.3% [7]. There is no evidence that the rate of incarceration is higher in UH treated with AS than the one observed with no medication; hence, it cannot be inferred that rapid narrowing of the hernia orifice could directly lead to incarceration. Although only the benefits of AS reported in Japan have been emphasized in observational studies, the complications associated with AS are rarely reported and may be underreported. In the future, the frequency of incarcerated hernias treated with AS should be investigated to prove that AS is accurately an effective and safe treatment in Japan where AS is mainly performed.

Additionally, our case shows that although the materials used in AS need to be replaced periodically in every case, it can lead to incarceration of the UH whenever replaced. Therefore, it is critical to perform replacement in as short a period as possible or be careful not to cause incarceration during replacement. Although AS has not yet been recommended in other countries [5], it could be an effective treatment for UH if performed properly, and it could be used worldwide in the near future. Although rare, clinicians should be fully aware that serious complications can occur in UHs during AS therapy. Furthermore, it is critical to instruct family members to consult medical institutions promptly when abdominal symptoms, such as abdominal pain and vomiting, appear during AS.

Limitation

Since this case report represents a single case, it is important to acknowledge the limitations in drawing definitive conclusions. The findings should be interpreted with caution, as the results may not be generalized to a large population. Further studies involving larger cases are warranted to validate this observation and establish more robust conclusions.

## Conclusions

AS for pediatric UH holds the potential to be a useful tool for earlier UH closure and a reduction in redundant skin of UH. However, AS does not necessarily eliminate the occurrence of incarcerated UH. In our case, AS treatment was performed appropriately. However, incarcerated UH occurred within a few tens of minutes during replacement, and it could have led to intestinal necrosis if left untreated for long. Fortunately, the patient was admitted in a short period of time after the onset of symptoms; hence, manual reduction was possible.

It is true that there are more serious complications other than incarceration. Above all, incarceration requires prompt recognition and treatment, and constant attention is required for infants with UH during AS treatment.
